# Millets and food security: a sustainable response to growing population needs

**DOI:** 10.3389/fpls.2026.1796931

**Published:** 2026-05-04

**Authors:** Tridisha Borah, Suresh Deka, Daisy Sharma

**Affiliations:** 1 Programme of Food, Nutrition and Dietetics, Assam down town University, Guwahati, Assam, India; 2 Programme of Microbiology, Assam down town University, Guwahati, Assam, India

**Keywords:** cultural acceptance, health benefit, millets, nutritional composition, nutritional security

## Abstract

The increasing lack and limitations of key cereals such as rice and wheat, aggravated by climate change, growing population, and declining agricultural sustainability, pose significant threats to global food and nutrition security. In this review, millets—often termed as “nutri-cereals”—appear as a viable alternative due to their resilience to adverse agro-climatic conditions, higher nutritional profile, and diverse health-promoting properties. This review synthesizes current knowledge on the potential of millets in enhancing food and nutritional security, focusing on their nutritional and antinutritional composition, processing methods, health benefits, and patterns of global and Indian production. *In vivo* and *in-vitro* evidence suggests that millet consumption helps in the prevention and management of chronic diseases such as diabetes, cardiovascular disorders, and certain cancers. Although enriched with essential nutrients and bioactive compounds, millets contain antinutritional factors that can be reduced through traditional and modern processing techniques. Despite these benefits, challenges to consumer acceptance persist, driven by cultural preferences, sensory limitations, and limited market penetration. Policy initiatives in India and globally reflect the increasing recognition of millets as a pathway to sustainable agriculture, livelihood security, and women’s empowerment. The integration of millets into mainstream diets, supported by processing technologies, public procurement, and awareness strategies, offers a sustainable solution to malnutrition while advancing progress toward the Sustainable Development Goals.

## Graphical Abstract

**Figure:**
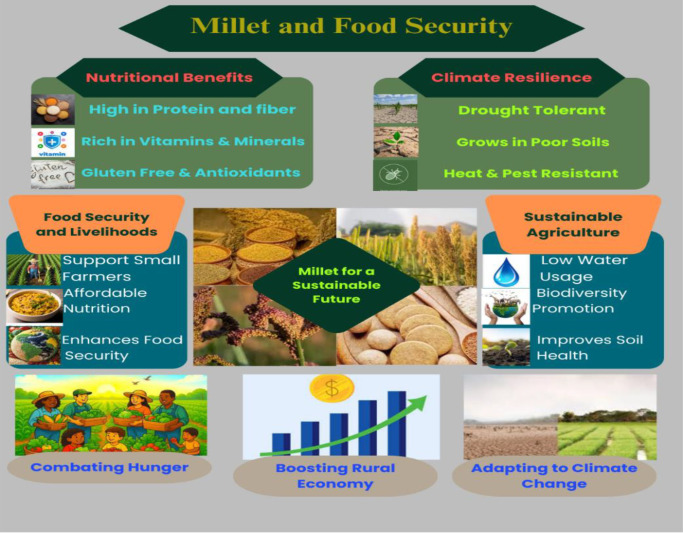

## 1 Introduction

Ensuring food security and combating hunger is one of the most significant challenges facing the world today. This crisis originated from factors such as inadequate nutrient consumption, reduced agricultural productivity, and continuing imbalance between supply and demand (Nithiyanantham et al., 2019). According to the *Global Report on Food Crises*, 2024, for the sixth consecutive year, the number of children who are severely food-insecure and malnourished has increased, due to conflicts, climatic shocks, and forced displacement in already vulnerable regions (Minot et al., 2024). An estimated 295 million people across 53 countries and territories faced hunger in 2024—an increase of 13.7 million from 281.3 million in 2023, representing a rise of approximately 4.9%. This figure corresponds to 22.6% of the total population across the 53 countries studied. UNICEF (2024) reported that about 38 million children under five in 26 crisis-affected regions are projected to suffer from acute malnutrition (UNICEF, 2024).

In order to address this challenge, it requires crops that are not only highly nutritious but also resilient to the very climatic and economic shocks exacerbating food insecurity. This is where traditional crops like millets offer a promising solution (Kane-Potaka et al., 2021). Their drought-tolerant nature, short growing season, and ability to thrive in poor soils make them uniquely suited to stabilize agricultural output in vulnerable regions, thereby helping to bridge the gap between nutritional demand and reliable food supply (Bandyopadhyay et al., 2017).

**Figure 1:**
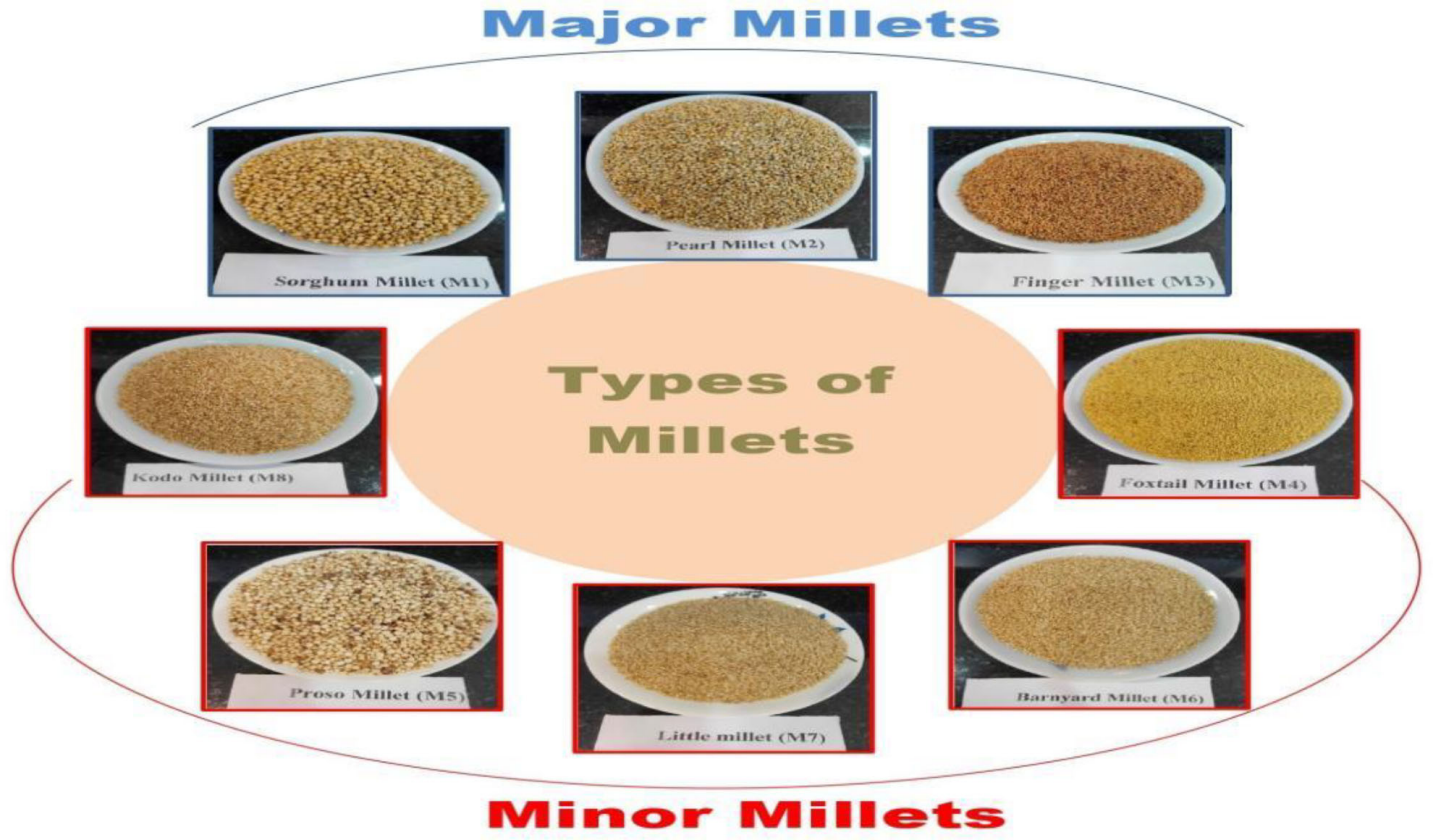
Types of millets.

Millets are a group of small-seeded cereal grains, often termed “nutri-cereals” due to their superior nutritional profile—characterized by high dietary fiber, essential minerals like iron and calcium, and a low glycemic index—compared to major cereals like wheat and rice (Anitha et al., 2021b; Bezbaruah and Singh, 2024). Their exceptional physiological and functional properties have led to them being considered functional or “super” foods (Muskan et al., 2025). Archaeological evidence suggests millets were among the earliest domesticated cereals. Especially, the discovery of well-preserved millet noodles at the Lajia archaeological site on the Huang He (Yellow River) provides direct material proof that millet-based food processing was technologically advanced in China approximately 4,000 years ago (Houyuan et al., 2005; Sharma et al., 2021). Millets are broadly divided into two groups, namely major and minor or small millets as illustrated in **Figure 1**. Sorghum millet (*Sorghum bicolor*), pearl millet (*Pennisetum typhoides*), and finger millet (*Eleusine coracana* L. Gaertn) belongs to major millets; on the other hand, foxtail millet (*Setaria italica*), kodo millet (*Paspalum scrobiculatum*), little millet (*Panicum sumatrense*), barnyard millet (*Echinochloa frumentacea*), and proso millet (*Panicum miliaceum*) are belong to minor or small millets (Maitra, 2020; Dey et al., 2022).

Historically, millets or nutri-cereals occupied a larger cultivation area than wheat and rice in many regions prior to the Green Revolution. The post-Green Revolution era, however, precipitated a dramatic decline in their cultivation. This shift was driven by policy incentives and the reallocation of irrigated lands to high-yielding varieties of economically favored staples like rice, wheat, and sugarcane (Birthal et al., 2013). Consequently, millet production has been increasingly marginalized to rainfed, low-input agro-ecological zones, including tribal and mountainous regions (Sukanya et al., 2023).

This decline is particularly concerning given the persistent challenge of malnutrition in developing countries, where diets heavily reliant on nutrient-deficient staple cereals contribute to hidden hunger. Achieving agricultural sustainability, therefore, requires a dual focus: not only increasing food production but also enhancing the nutritional density of food systems. In this context, millets are a compelling solution; their high nutritional content and exceptional resilience to diverse and harsh agro-climatic conditions make them a strategic crop for improving nutritional security, especially among vulnerable populations. Promoting their cultivation and consumption is a critical step toward simultaneously achieving health and sustainability goals (Maitra and Shankar, 2019).

Despite the growing body of literature on millets, existing reviews are often limited in scope, primarily focusing on isolated aspects such as nutritional composition, health benefits, or agronomic performance. There is a lack of comprehensive analyses that position millets explicitly within the broader context of food security and sustainable responses to increasing population demands. Therefore, the present review aims to provide a holistic and critical synthesis of the role of millets in addressing food and nutritional security challenges. The review integrates current knowledge on their nutritional and antinutritional composition, processing technologies, and health benefits with emerging insights into consumer behavior, adoption barriers, and policy interventions. It adopts an interdisciplinary and global perspective, including Sub-Saharan Africa, while highlighting key research gaps and practical challenges. The review also proposes a focused research agenda to support the integration of millets into sustainable and climate-resilient food systems.

## 2 Methodology

This study was conducted as a narrative review aimed at synthesizing existing knowledge on the role of millets in food security and sustainable nutrition. The methodology employed in this review was adapted from previously established protocols, with minor modifications to suit the scope of the study (Guiné et al., 2021). A structured literature search was carried out to identify relevant scientific publications, reports, and policy documents related to millets. The process involved applying appropriate filters, screening titles and abstracts, and using clearly defined inclusion and exclusion criteria to ensure the relevance and reliability of the selected studies. This approach enabled a comprehensive overview of the current literature on the nutritional composition, processing, health benefits, and policy aspects of millets.

### 2.1 Article identification

Relevant articles are usually identified from multiple databases such as Science Direct, Google Scholar, PubMed, Web of Science and Research Gate. The literature search was conducted using predefined keywords such as millets, nutritional and health benefits of millets, effects of processing technologies on millets, socio-economic aspects of millets, consumer acceptance, and geographical distribution, filtering articles by publication date; limiting the search to articles published in English language; and selecting only peer-reviewed articles, clinical trials, reviews, or specific study designs relevant to the topic.

### 2.2 Screening process

The initial screening involved evaluating the titles and abstracts of retrieved articles to assess their relevance to the review objectives. Articles deemed potentially relevant were subsequently subjected to a comprehensive full-text review, during which their suitability for inclusion was determined based on predefined, detailed eligibility criteria.

### 2.3 Inclusion criteria

Studies were included if they directly addressed the objectives of this review and met defined methodological standards. Both original research articles and review papers focusing on millets were considered eligible for inclusion.

### 2.4 Exclusion criteria

Articles that were not directly related to the research topic or question, as well as studies lacking sufficient methodological or analytical detail, were excluded. In addition, non–peer-reviewed publications, opinion pieces, and editorials were omitted from consideration.

By employing this systematic approach, the final selection of articles was ensured to be robust, relevant, and aligned with the objectives of the manuscript.

## 3 Environmental, socio-economic sustainability and integration of millets into food systems

Millets significantly enhance environmental sustainability in food systems due to their resilience, low resource requirements, and nutritional benefits. These ancient grains, including varieties like sorghum and pearl millet, thrive in harsh conditions, requiring less water and fertilizers compared to conventional crops such as, rice and wheat, making them ideal for climate-resilient agriculture (Ashoka et al., 2023; Dixit and Ravichandran, 2024; Pandey et al., 2025). Their cultivation supports biodiversity and traditional mixed cropping systems, which promote soil health and reduce pest pressures (Bhushan et al., 2024; Pandey et al., 2025). Nutritionally, millets are rich in essential minerals, vitamins, and dietary fiber, contributing to improved health outcomes and reduce the risk of chronic diseases. Furthermore, promoting millet consumption can empower rural economies and enhance food security, aligning with Sustainable Development Goals by fostering sustainable agricultural practices and improving dietary diversity (Bhushan et al., 2024; Dixit and Ravichandran, 2024; Pandey et al., 2025). Thus, millets represent a sustainable solution to the challenges posed by climate change and food insecurity.

## 4 Role of millets in ensuring nutritional security

The achievement of food and nutrition security is contingent upon universal and reliable access to sufficient, safe, and culturally acceptable food. However, access alone is insufficient; it must be coupled with an enabling environment of adequate sanitation, health services and care to facilitate optimal food utilization and overall well-being. Nutrition security forms an integral part of overall food security, as adequate nutrition involves more than just meeting caloric needs. It requires the availability and accessibility of a diverse and balanced diet that satisfies the specific nutritional demands of all population groups- men, women and children alike (Hwalla et al., 2016a). As outlined by UNICEF, the three primary pillars influencing nutritional security include reliable access to adequate and nutritious food, appropriate care-giving and feeding practices, and access to sanitation and healthcare services across all segments of the population (Wüstefeld, 2013a).

In fact, achieving nutrition security requires the combined presence of adequate food, healthcare, and care-giving. Therefore, it cannot be realized without ensuring sufficient food availability for households (FAO, 2009a). The global issue of nutritional insecurity is especially severe among populations that predominantly consume cereal-based diets, which are often lacking in vital micronutrients. In regions characterized by arid and drought-prone climates across Asia and Africa, millets act as key staple foods, ranking just after cereals (Vinoth and Ravindhran, 2017a). Nutritional insecurity remains a major concern among communities whose diets are heavily dependent on carbohydrate-rich foods with limited access to essential micronutrients such as vitamins and minerals. Therefore, fortifying micronutrients in carbohydrate rich diets represents a viable strategy to enhance dietary diversity and reduce the risk of malnutrition. Millets, being rich in essential micronutrients, dietary fiber, bioactive compounds, and vitamins with various therapeutic properties, hold significant potential as alternative staple grains. Their integration into regular diets can contribute substantially to promoting food and nutrition security across diverse geographical regions (Konapur et al., 2014a; Kumar et al., 2018a).

## 5 Nutritional composition of millets

In terms of nutrition, millets are comparatively better than most commonly consumed cereals (Hwalla et al., 2016b). Millets are important in promoting nutritional security because they are rich in calories and protein (Wüstefeld, 2013b). Additionally, millets are an excellent source of essential micronutrients, including B-complex vitamins such as niacin, riboflavin, and thiamine, which are necessary for proper metabolic function and overall health (FAO, 2009b). Millets offer approximately 320 to 370 kilocalories per 100 grams and consist of about 65-75% carbohydrates. While millet proteins lack sufficient lysine and threonine, they are notably high in methionine, making them a valuable protein source. Additionally, millets are packed with essential micronutrients and phytochemicals (Vinoth and Ravindhran, 2017b; Kumar et al., 2018b). Millets are rich in a range of health-promoting phytochemicals, including phytosterols, polyphenols, phytocyanins, lignins, and phytoestrogens (Konapur et al., 2014b). Among all the millets, finger millet records the highest carbohydrate content at 72.0-72.6 g/100 g, followed by proso millet (63.8-70.4 g), barnyard millet (55.0-65.5 g), pearl millet (61.8-67.5 g), kodo millet (65.9-78.2 g), and little millet (67.0 g), while the lowest is observed in foxtail millet (60.9-63.2 g) (**Table 1**). For protein, proso millet contains the highest amount at 12.5 g/100 g, followed by foxtail millet (11.2–12.3 g), pearl millet (10.9–11.6 g), sorghum (10.4-10.6 g), and barnyard millet (6.2 g), whereas the lowest protein is found in finger millet (7.3-7.7 g) and kodo millet (6.8-8.3 g) (**Table 1**). Millet grains contain a moderate amount of fat, with pearl millet showing the highest content (5.0-5.43 g/100 g). This is followed by little millet (4.7 g/100 g) and foxtail millet (4.3 g/100 g), while kodo millet contains the least (0.5 g/100 g) (**Table 1**). These fats are predominantly unsaturated fatty acids, such as linoleic and oleic acids, which contribute to the nutritional quality and health-promoting properties of millets (Kumar et al., 2024; Pandey et al., 2024a). With respect to energy, pearl millet again has the highest value at 348–361 kcal/100 g, followed by foxtail millet (331–351 kcal), kodo millet (345 kcal), finger millet (336 kcal), and sorghum (329–334 kcal), while the lowest is observed in barnyard millet (300 kcal) (**Table 1**). For crude fiber, barnyard millet ranks highest with 9.8 g/100 g, followed by kodo millet (9.0 g) and little millet (7.6 g), whereas the lowest fiber content is found in pearl millet (1.2-2.3 g) (**Table 1**). In terms of dietary fiber, millets offer nearly twice the amount found in rice and are comparable to whole wheat. They contain a mix of soluble fibers- such as β-glucans, arabinoxylans, and pectins- as well as insoluble fibers like cellulose and hemicellulose (Longvah et al., 2017). Additionally, millets are an excellent source of micronutrients, as finger millet is by far the richest in calcium with 344–350 mg/100 g, while the lowest content is reported in little millet (12–17 mg) (**Table 1**). For iron, little millet records the maximum value of 9.3 mg/100 g, followed by pearl millet (8.0 mg) and barnyard millet (5.0 mg), whereas kodo millet shows the least at 0.5 mg/100 g. In terms of zinc, little millet again contains the highest amount (3.5 mg/100 g), while the lowest is found in kodo millet (1.5 mg/100 g) (**Table 1**). For phosphorus, pearl millet provides the maximum (296 mg/100 g), followed by foxtail millet (290 mg) and finger millet (283 mg), with the lowest content in kodo millet (188 mg/100 g). Regarding sodium, pearl millet has the highest content at 10.9 mg/100 g, while the lowest value is reported in sorghum (2 mg/100 g) (**Table 1**). They also provide essential vitamins like thiamine (0.25-0.59 mg/100 g), niacin (0.86-4.50 mg/100 g), and riboflavin (0.10-0.28 mg/100 g) (Konapur et al., 2014b; Nithiyanantham et al., 2019; Baduni et al., 2024; Gahalawat et al., 2024).

**Table 1:** Nutritional composition of millets (Per 100g).

Component		Sorghum millet	Pearl millet	Finger millet	Foxtail millet	Proso millet	Barnyard millet	Little millet	Kudo millet
Nutritional Compositi-on Of Millets (Per 100gm)	Moisture(g)	8.97-12.4	8.97-12.4	13.1	11.2	11.9	–	–	–
	Ash(g)	1.37-1.6	1.37-2.2	2.7	2.7-3.3	1.9-3.1	4.4	1.5	0.6
	Protein (g)	10.4-10.6	10.96-11.6	7.3-7.7	11.2-12.3	12.5	6.2	7.7	6.8-8.3
	Carbo-Hydrate (g)	67.68-72.6	61.78-67.5	72.0-72.6	60.9-63.2	63.8-70.4	55-65.5	67.0	65.9-78.2
	Total Fat(g)	1.7-3.43	5-5.43	1.3-1.5	4.0-4.3	1.1-3.5	2.2	4.7	0.5
	Energy (Kcal)	329-334.13	347.99-361	336	331-351	341	300	329	345
	Crude Fiber(g)	1.6-2.0	1.2-2.3	3.6	8.0	2.2	9.8	7.6	9
Minerals (Per100g)	Calcium(mg)	13-25	42.0	344-350	31	14	20-21	12-17	27-31
	Iron(mg)	4.1	8.0	3.9	2.8	0.8	5.0	9.3	0.5
	Zinc(mg)	1.7	3.1	2.3	2.4	1.4	2.6	3.5	1.5
	Phosphorous(mg)	222	296	283	290	206	280	220	188
	Sodium(mg)	2	10.9	11	4.6	8.2	–	–	–
Vitamins (Per100g)	Niacin(mg)	3.7-5.19	1.11-2.3	0.80-1.1	0.55-3.2	2.3-4.54	0.10	3.2	0.09
	Thiamin(mg)	0.33	0.33	0.42	0.59	0.2	–	–	–
	Riboflavin(mg)	0.096	0.25	0.19	0.11	0.18	–	–	–

Source (Suma and Urooj, 2014; Morah and Etukudo, 2017a; Sharma and Niranjan, 2018a; Gowda et al., 2020; Yousaf et al., 2021):

However, the nutritional value of millets is not determined solely by their nutrient composition but also by the bioavailability of these nutrients during digestion. The presence of anti-nutritional compounds such as phytates, tannins, and certain polyphenols can bind minerals and proteins, forming complexes that reduce their absorption in the gastrointestinal tract. As a result, although millets contain considerable amounts of minerals such as iron, zinc, and calcium, their actual absorption may be limited without appropriate processing. Therefore, suitable processing techniques are essential to improve nutrient digestibility and enhance mineral bioavailability in millet-based foods.

## 6 Processing of millets

Processing plays a crucial role in improving the digestibility, bioavailability, and overall nutritional utilization of millets. Raw millets are not directly edible and require processing to remove inedible components such as husk and to improve their palatability, shelf stability and nutrient accessibility (Konapur et al., 2014b). Moreover, processing helps to reduce anti-nutritional factors that limit nutrient absorption, thereby enhancing the bioavailability of minerals, proteins, and bioactive compounds. There are many ways to process millets, including dehusking, milling, soaking, sprouting, fermenting and cooking. These methods change the foods physical, functional, and nutritional qualities (Amadou et al., 2014). Processing techniques fall into two main groups. The first, primary processing, involves basic steps like cleaning, soaking, dehulling, and milling into flour to eliminate the outer layer and antinutrients. The second, secondary processing, converts these ingredients into convenient products such as “ready-to-cook” or “ready-to-eat” items by using methods like extrusion, flaking, or baking (Konapur et al., 2014b). Traditional methods like roasting, steaming, and fermentation also help create shelf-stable, digestible grains with better texture, flavor, and nutrition (Rani et al., 2018a). The nutritional impact of these methods varies with the types of millet and its processing techniques. Germination usually improves protein, fiber, minerals, and bioactive compounds while lowering fat and carbohydrates. This is evident with foxtail, pearl, kodo, and proso millets (Deshpande et al., 2015; Sharma and Niranjan, 2018b). Soaking and cooking can increase the bioavailability of minerals like zinc and iron and improve antioxidants or resistant starch. However, these methods might also lead to losses in protein, fat, and heat-sensitive compounds due to leaching and thermal breakdown (Sharma et al., 2015). Fermentation boosts protein digestibility, starch quality, and sugar levels, while reducing carbohydrate and fat content (Bello et al., 2017). Cooking methods can enhance antioxidant activity and functional attributes such as oil absorption, but they might also decrease fiber, fat, calcium, or polyphenols, depending on the variety (Obadina et al., 2017). Overall, the nutritional benefits of millets depend not only on their nutrient composition but also on the bioavailability and effective absorption of these nutrients in the human body. Although millets are rich in essential macronutrients, minerals, vitamins, and bioactive compounds, the presence of anti-nutritional factors such as phytates and tannins can limit the absorption of certain minerals, particularly iron, zinc, and calcium. Processing techniques including soaking, germination, fermentation, and thermal treatments play a significant role in reducing these anti-nutritional compounds and improving nutrient digestibility and mineral bioavailability. However, processing can lead to both beneficial and adverse changes in nutrient profiles depending on the method and intensity applied. Extensive research indicates that the selection of appropriate processing techniques is crucial for maximizing nutrient bioavailability and enhancing the functional properties of millets. This not only improves their nutritional utilization but also facilitates their incorporation into traditional diets as well as modern health-oriented food products (Konapur et al., 2014b; Iyabo et al., 2018). The summarized effects of various mechanical and traditional processing methods on millet nutritional quality are presented in **Table 2**.

**Table 2:** The effects of different processing methods on various millets.

Millet type	Processing method	Positive effects (↑)	Negative effects (↓)	References
Foxtail Millet	Germination	↑ Protein, fiber, minerals, phenolics	↓ Fat	(Chinenye et al., 2017; Rani et al., 2018b)
	Soaking + Cooking	↑ Bioavailable Zn & Fe	↓ Protein, Fe, Zn	(Sharma et al., 2015)
	Fermentation	↑ Protein, starch quality	↓ Carbohydrates	(Sharma et al., 2015; Bello et al., 2017)
Pearl Millet	Germination	↑ Iron, Ca	↓ Fat, ash	(Sharma and Niranjan, 2018b)
	Malting	↑ Protein, fiber	↓ Carbs, fat, amino acids	(Morah and Etukudo, 2017b; Sharma et al., 2017)
	Soaking	↑ Protein, fat, fiber, Ca	↓ Carbs, minerals	(Pradeep and Sreerama, 2015)
	Fermentation	↑ Protein, sugars	↓ Lipids, carbs	(Pandey et al., 2024b)
	Cooking	↑ Polyphenols, flavonoids, resistant starch	↓ Polyphenols (PC)	(Mishra et al., 2022)
Kodo Millet	Germination	↑ Protein, fiber, minerals	↓ Carbohydrates	(Han et al., 2018)
	Cooking	↑ Resistant starch, protein, oil absorption	↓ Fiber, fat, Ca	(Iyabo et al., 2018)
Proso Millet	Germination	↑ Protein & mineral bioavailability	–	(Sudhakar et al., 2015)
	Cooking	↑ Carbs, antioxidants	↓ Fat; Protein varies	(Subba Rao and Muralikrishna, 2002)
Little Millet	Cooking	↑ Carbs	↓ Fat; Protein varies	(Subba Rao and Muralikrishna, 2002)

*↑, Increase/Improvement; *↓, Decrease/Reduction; * PC, Phenolic compounds; * -: No significant negative effect reported.

## 7 Functional and therapeutic benefits of millets

The nutritional transition in India, accelerated by urbanization, has resulted in decreased consumption of traditional cereals like millets and a concurrent rise in the intake of animal products, refined sugars, and fats (Saleh et al., 2013a; Udeh et al., 2017). This dietary shift is a key factor in the increasing global prevalence of non-communicable diseases, which are responsible for an estimated 71% of all deaths worldwide.

These modern diets promote oxidative stress, a condition characterized by an excess of reactive oxygen species (ROS) that causes cellular damage to DNA. This process is a significant mechanism in the development of aging, diabetes, cancer, and various cardiovascular, neurological, and inflammatory disorders. Antioxidants, whether endogenous or obtained from dietary sources, counteract this by neutralizing ROS and supporting immune function. Millets are a rich source of such natural antioxidants and exhibit potent free radical scavenging activity. Consequently, their consumption can effectively mitigate oxidative stress and reduce the risk of degenerative diseases mediated by free radicals (Saleh et al., 2013a).

### 7.1 Antioxidant potential of millets and associated bioactive compounds

Millets exhibit significant antioxidant properties due to their rich composition of bioactive compounds, including phenolic acids, flavonoids, tannins, lignans, and phenolic diterpenes. Various millet types such as foxtail, barnyard, kodo, proso, finger, and little millet contain both soluble and insoluble polyphenols that contribute substantially to their antioxidant potential. Among these, finger millet (ragi) is particularly rich in phenolic compounds such as ferulic acid, caffeic acid, and quercetin, while foxtail millet contains catechins and vanillic acid. Pearl millet additionally provides lignans such as secoisolariciresinol diglucoside, which are associated with antioxidant and anti-inflammatory properties.

*In vitro* studies have consistently demonstrated strong antioxidant activity in millets, primarily through free radical scavenging mechanisms such as single electron transfer and hydrogen donation, which neutralize reactive oxygen species (ROS) (Shobana et al., 2009; Kim et al., 2011; Chaudhary and Mudgal, 2020). These antioxidant capacities are commonly evaluated using assays such as DPPH and FRAP (Dhanorya et al; Ofosu et al., 2021). Methanolic extracts of different millet varieties have also shown considerable antioxidant potential, as evidenced by ferric reducing antioxidant power and oxygen radical absorbance capacity assays, with pigmented varieties such as sweet sorghum exhibiting comparatively higher activity (Abeysekera et al., 2022). Finger millet, in particular, has been reported to possess high phenolic and flavonoid content, contributing to its strong antioxidant capacity (Ofosu et al., 2021).

*In vivo* studies further support these findings by demonstrating the role of millet-based diets in enhancing antioxidant defense systems. For example, consumption of foxtail millet-based composite flour has been associated with increased activity of antioxidant enzymes such as superoxide dismutase and glutathione peroxidase, along with reduced malondialdehyde levels, indicating mitigation of oxidative stress (Lin et al., 2023). Additionally, processing techniques such as sprouting and the development of microgreens have been shown to enhance antioxidant potential by increasing the concentration of secondary metabolites (Durairajan et al., 2024).

Overall, the available evidence indicates that millets possess considerable antioxidant potential. However, most findings are derived from *in vitro* and animal-based studies, and therefore, well-designed human clinical trials are necessary to confirm these effects and establish their relevance to human health.

### 7.2 Antidiabetic potential of millets

Millets are increasingly recognized for their potential role in the prevention and management of diabetes due to their low glycemic index (GI), high dietary fiber content, and rich composition of nutrients and bioactive compounds. Millets such as sorghum and foxtail millet have been reported to exhibit lower GI values compared to staple cereals like rice and wheat, thereby slowing glucose release into the bloodstream and improving glycemic response (Anitha et al., 2021a; Mansoria and Singh, 2023). Regular consumption of whole grains, including millets, has also been associated with a reduced risk of type 2 diabetes (McKenney, 2004; Miller et al., 2011; Chait and Eckel, 2016).

Findings from *in vivo* studies, including both human and animal models, indicate that millet-based dietary interventions can improve glycemic control. Reductions in fasting blood glucose, postprandial glucose levels, and glycated hemoglobin (HbA1c) have been reported following millet consumption (Anitha et al., 2021a). For instance, a 12-week intervention study by Ren et al. (2022) demonstrated that daily intake of 50 g of foxtail millet in individuals with impaired glucose tolerance significantly reduced fasting and 2-hour postprandial blood glucose levels and improved insulin sensitivity (Jali et al., 2012). Similarly, experimental studies in diabetic animal models have shown that millet-based formulations, such as finger millet–enriched probiotic fermented milk, can improve lipid profiles, enhance hepatic enzyme activity, and provide protective effects on pancreatic tissues. However, the variability in study design and scale should be considered when interpreting these findings.

*In vitro* studies provide mechanistic insights into these observed effects. The high content of dietary fiber, resistant starch, and polyphenols in millets has been shown to reduce starch digestibility and delay glucose release. These components may also inhibit carbohydrate-digesting enzymes and contribute to improved glucose metabolism. Additionally, the presence of magnesium and antioxidant compounds further supports their role in enhancing insulin sensitivity and reducing oxidative stress (VP et al., 2024; Mahendran and Krishnaja, 2025).

Overall, the available evidence suggests that millets possess promising antidiabetic properties. However, further well-designed, large-scale human clinical trials are required to confirm these effects and establish clear dietary recommendations.

### 7.3 Role of millets in cardiovascular health and lipid profile modulation

Millets are increasingly recognized for their potential role in the prevention and management of cardiovascular diseases (CVDs), which are closely associated with metabolic risk factors such as elevated low-density lipoprotein (LDL) cholesterol and triglycerides. Dietary strategies aimed at reducing LDL levels are considered effective approaches for lowering CVD risk (Nishizawa et al., 1990; Chandrasekara and Shahidi, 2011; Joshi and Srivastava, 2021). Millets are rich in nutrients such as dietary fiber, magnesium, and niacin, which contribute to improved lipid metabolism and overall cardiovascular health (Saleh et al., 2013b).

*In vivo* studies indicate that millet-based dietary interventions can positively influence lipid profiles. Several intervention studies have reported reductions in LDL cholesterol and triglyceride levels, along with improvements in high-density lipoprotein (HDL) levels following the consumption of millet-based foods (Graf and Eaton, 1990; Thompson, 1993). However, some studies have noted improvements in glycemic parameters without significant changes in HDL levels (Shan et al., 2014). Further support is provided by a systematic review and meta-analysis conducted by Anitha et al., which included 19 human studies and demonstrated that regular millet consumption over periods ranging from 21 days to 4 months significantly reduced total cholesterol, triglycerides, LDL-C, and very-low-density lipoprotein (VLDL-C), while showing modest increases in HDL-C (Anitha et al., 2021a). These findings highlight the potential of millets as a functional dietary approach for improving lipid profiles and managing conditions such as hyperlipidemia and obesity.

### 7.4 Anticancer potential of millets

Millets contain a wide range of bioactive compounds, including phenolic acids, flavonoids, tannins, and dietary fiber, which have been associated with potential anticancer properties (Amadou et al., 2013a; Zhang et al., 2020a). These compounds are known to modulate various molecular pathways involved in carcinogenesis, including oxidative stress, inflammation, and cell proliferation.

*In vitro* studies have demonstrated that millet-derived bioactive compounds can inhibit cancer cell proliferation and induce apoptosis in different cancer cell lines. For instance, polyphenol-rich extracts from pearl millet have shown significant cytotoxic activity against multiple breast cancer cell lines, including triple-negative, ER-positive, and HER2-positive types. These extracts were reported to induce apoptotic cell death, reduce metabolic activity, and exhibit minimal toxicity toward non-tumorigenic cells. Bioactive compounds such as apigenin, caffeic acid, and luteolin are considered to contribute to these effects, and approaches such as microencapsulation have been suggested to enhance their bioavailability and therapeutic potential (Hajri et al., 2024). Similarly, a 35 kDa fibroin-modulator-binding protein (FMBP) isolated from foxtail millet has been shown to suppress colon cancer cell growth by inducing G1 phase arrest and activating apoptotic pathways (Warisa et al., 2025a). Additional studies have reported that polyphenol-rich extracts from millets exert anticancer effects by inhibiting cancer cell proliferation and promoting programmed cell death.

*In vivo* studies further support these findings by demonstrating the effects of millet-based interventions in animal models. Experimental studies have shown that foxtail millet supplementation can reduce colitis-associated colorectal cancer by inhibiting key signaling pathways such as STAT3, which is involved in tumor growth and inflammation (De Silva Perera and Inder, 2021a). In a mouse model, dietary inclusion of foxtail millet significantly suppressed tumor formation, reduced inflammatory responses, and modulated gut microbiota and their metabolites (Zhang et al., 2020b). Furthermore, studies involving xenograft models have reported that foxtail millet bran-derived peroxidase (FMBP) can selectively bind to glucose-regulated protein 78 (GRP78), leading to inhibition of the STAT3 pathway, increased intracellular reactive oxygen species (ROS), and induction of apoptosis in cancer cells, while exhibiting minimal toxicity toward normal cells (Amadou et al., 2013b; Shan et al., 2022).

Overall, these findings suggest that millet-derived bioactive compounds possess promising anticancer potential through multiple mechanisms, including modulation of signaling pathways, induction of apoptosis, and reduction of inflammation. However, most of the available evidence is limited to *in vitro* and animal studies, and therefore, well-designed human clinical trials are required to validate these effects and determine their clinical relevance.

### 7.5 Millet for celiac disease

Celiac disease is a genetically susceptible problem triggered by the consumption of gluten. As the millets are gluten free, they may help in reducing the celiac disease by reducing the irritation caused by the common cereal grains which contain gluten (Zhang et al., 2020b).

### 7.6 Evidence from human clinical studies and existing research gaps

Although a growing number of experimental studies have highlighted the potential health benefits of millets, the availability of well-designed human clinical trials remains limited. Existing human studies have mainly focused on metabolic outcomes such as glycemic control and lipid metabolism. For example, clinical interventions have reported that regular consumption of foxtail millet or other millet-based foods can improve fasting blood glucose levels, postprandial glucose response, and insulin sensitivity in individuals with impaired glucose tolerance. Similarly, some dietary intervention studies and meta-analyses have indicated that millet consumption may contribute to improvements in lipid profiles, including reductions in total cholesterol, triglycerides, and low-density lipoprotein (LDL) cholesterol. Despite these promising findings, most studies have relatively small sample sizes, short intervention durations, and variations in millet species, processing methods, and dietary protocols. Moreover, human clinical evidence evaluating other potential health benefits of millets, particularly their antioxidant, anti-inflammatory, and anticancer effects, is still scarce. Therefore, large-scale, long-term randomized controlled trials are required to better understand the bioavailability of millet-derived bioactive compounds and to establish clear dietary recommendations for the prevention and management of chronic diseases.

## 8 Major challenges limiting millet production, processing, and consumption in India

A summary of the major challenges limiting millet production, processing, and consumption in India is provided in **Table 3**.

**Table 3:** Major challenges limiting millet production, processing, and consumption in India.

Category	Key issue	Remarks	Reference
Cultivation	Decrease area under millet cultivation	The area devoted to millet cultivation has declined substantially due to low yield potential, labor-intensive post-harvest operations, limited mechanization, and inadequate value addition and market incentives.	(Amadou et al., 2013b; Orr et al., 2020; Shah et al., 2023)
Productivity	Low and stagnant yields	Production trends indicate a decline in sorghum, stagnation in pearl millet, and minimal yield improvement in finger millet and other small millets over the past decade.	
Awareness	Poor consumer knowledge	Inadequate knowledge regarding the nutritional, functional, and health-promoting properties of millets has resulted in weak consumer demand.	
Economic	Higher consumer price	Compared to major cereals rice and wheat, millets are often more expensive, limiting their affordability for low-income population groups.	
Availability	Limited retail presence	Inadequate distribution in traditional markets and insufficient penetration in modern retail and e-commerce platforms restrict accessibility.	
Consumer acceptance	Taste and convenience barriers	Consumer preference is diminished due to lack of product variety, convenience foods, and familiarity with millet-based diets.	
Agronomy	Low farmer profitability	Farmers often perceive millet cultivation as economically less profitable due to yield uncertainty, price volatility, and inadequate market support.	
Market competition	Dominance of rice and wheat	Strong policy backing, consumer familiarity, and well-established supply chains favor rice and wheat, marginalizing millets in the food system.	
Policy	Inadequate institutional support	Insufficient policy incentives, procurement mechanisms, and long-term promotional strategies constrain millet sector growth.	
Processing	Decentralized and complex processing	Variability in grain size, shape, hardness, husk adhesion, and regional production conditions complicates standardized processing and mechanization.	
Storage	Poor shelf life of processed millets	Enzymatic activity, lipid oxidation, moisture sensitivity, and rapid rancidity reduce the shelf stability and sensory quality of millet-based products.	

## 9 Consumer perception, acceptance, and adoption barriers of millet-based foods

Consumer acceptance of millet-based foods is multidimensional, involving not only sensory attributes but also behavioral, socio-cultural, and market-related factors. Several studies have demonstrated that consumers generally perceive millets as nutritionally rich, high-quality, and beneficial for health, which positively influences their consumption intentions (Kaur and Banga, 2025). Health consciousness, environmental sustainability, and perceived product quality are key drivers encouraging consumers to include millets in their diet (Kaur and Banga, 2025). Additionally, social influence, peer networks, and exposure through awareness campaigns play a significant role in shaping consumer behavior and facilitating the dietary shift toward millets, particularly among urban populations (Singh and Vemireddy, 2023).

Despite these favorable perceptions, the actual adoption of millet-based foods remains relatively low due to multiple constraints. Lack of awareness regarding specific health benefits, limited availability in local markets, higher price compared to conventional cereals, and inadequate product visibility act as major barriers to consumption (Singh and Vemireddy, 2023; Kaur and Banga, 2025). Furthermore, household food habits, taste preferences, and unfamiliarity with millet-based recipes restrict their regular inclusion in daily diets (Kaur and Banga, 2025). Market-related challenges such as insufficient branding, lack of attractive packaging, and limited product diversification also hinder wider consumer acceptance and commercialization of millet-based products. Evidence from product development and consumer studies suggests that acceptance can be significantly improved when millets are incorporated into familiar food formats without compromising sensory properties. For example, millet-based composite products such as biscuits and blended flour foods have demonstrated good acceptability in terms of taste, texture, and overall preference among consumers (Chinma et al., 2022; Kariuki et al., 2025). Moreover, providing consumers with information regarding the nutritional and health benefits of millet-based foods has been shown to enhance their willingness to purchase and consume such products (Kariuki et al., 2025).

Overall, while consumer attitudes toward millets are increasingly positive, translating this perception into sustained consumption requires addressing key barriers related to awareness, affordability, accessibility, and product innovation. Strategic interventions focusing on nutrition education, improved market availability, and development of convenient and appealing millet-based products are essential to enhance their widespread acceptance.

## 10 Millets for India’s future (policy and practice)

The Indian government has implemented a comprehensive strategy to promote millet farming, aiming to improve food security and tackle climate change. This includes national initiatives like the National Food Security Mission (NFSM), which increases production through seed distribution and training. The National Mission for Sustainable Agriculture (NMSA) also supports sustainable farming practices. Additional support comes from programs like the Pradhan Mantri Krishi Sinchai Yojana (PMKSY) for drought-prone areas and the Rashtriya Krishi Vikas Yojana (RKVY), which focuses on productivity and access to credit. To strengthen the value chain, policies such as the National Food Processing Policy promote value addition. The PM-AASHA scheme ensures a minimum support price. These initiatives are backed by state-level programs, including Karnataka’s Millets Promotion and Development Program (MPDP), as well as other efforts like the Production Linked Incentive (PLI) Scheme, inclusion in the Public Distribution System (PDS), and dedicated research (Ministry of Food Processing Industries, Government of India, 2022) (Warisa et al., 2025b). A promising way to boost both consumption and supply is to integrate millets into major national nutrition schemes such as the Integrated Child Development Services (ICDS) and the Midday Meal Program. Adding millets to these programs in appealing forms can greatly enhance children’s nutritional intake and raise early awareness of their health benefits (De Silva Perera and Inder, 2021b). At the same time, better farmer support through improved price mechanisms, low-cost financing, and easier access to credit can secure a steady supply for these initiatives. However, the success of these varied efforts faces challenges like weak infrastructure, limited resources, fragmented market chains, and the difficulties of reaching smallholder farmers in remote areas. For a more effective implementation, it is also important to protect traditional cultivation knowledge, encourage farmer exchanges, and safeguard indigenous seed varieties. This combined approach of stimulating demand through public programs while strengthening the agricultural supply chain can advance nutritional security, promote sustainable agriculture, and empower rural livelihoods.

Beyond India, Sub-Saharan Africa offers critical policy insights into the promotion of millets as climate-resilient and nutritionally significant crops. Across the region, where millets are traditional staples, countries such as Nigeria, Niger, and Mali have integrated millets into national food security and climate-resilient agriculture strategies. At the regional level, coordinated initiatives led by organizations including the Food and Agriculture Organization, CIMMYT, Bill & Melinda Gates Foundation, African Union, and the International Crops Research Institute for the Semi-Arid Tropics have focused on addressing structural bottlenecks in millet cultivation through improvements in genetic resources, farmer training, and value chain development (African Union Development Agency–NEPAD, 2020). National strategies, such as those implemented in Senegal, further emphasize increasing productivity through improved seed systems, expansion of cultivated areas, and enhanced access to agricultural inputs and infrastructure. In addition, large-scale initiatives such as the Africa Dryland Crops Improvement Network and the Vision for Adapted Crops and Soils (VACS) project highlight a growing policy shift toward research-driven, climate-adaptive agriculture tailored to dryland ecosystems. The global recognition of millets through the International Year of Millets 2023 has further accelerated policy attention and international collaboration, reinforcing the role of millets in addressing food security and climate resilience challenges, particularly in the Sahel region where their adaptability to arid and semi-arid conditions is crucial. Moreover, increasing emphasis on effective communication and consumer awareness is helping to reposition millets from “neglected crops” to integral components of sustainable food systems. However, persistent challenges—including limited infrastructure, gaps in research translation, and the need for locally driven innovation—continue to constrain progress. Therefore, a comprehensive policy approach integrating production enhancement, research and innovation, value chain strengthening, and consumer-oriented strategies is essential to fully realize the potential of millets in Sub-Saharan Africa and beyond (Revolutionizing agriculture in Sub-Saharan Africa: A bright future for millet cultivation).

In addition, Sudan presents an important case for understanding millet-based policy interventions in semi-arid and resource-constrained regions. Millet, being a climate-resilient and nutritionally rich crop, holds significant potential for enhancing food security and sustainable agriculture in Sudan. Policy options in this context emphasize improving production through the development of high-yielding, drought-tolerant varieties, strengthening agricultural extension services, and promoting sustainable land and water management practices. Equally important is the strengthening of millet value chains through investments in processing infrastructure, improved market linkages, and consumer awareness initiatives to enhance demand and economic viability. Furthermore, promoting research and innovation, building climate resilience through adaptive farming strategies, and ensuring policy coordination across sectors are critical for long-term sustainability. Legislative support, including subsidies, price support mechanisms, and infrastructure development, can further incentivize millet cultivation and commercialization. However, the effectiveness of these policies may be constrained by challenges such as limited financial resources, weak infrastructure, low consumer awareness, and socio-economic barriers including land tenure issues and limited access to credit. Therefore, a holistic and context-specific policy framework integrating production, market, and consumer-oriented strategies is essential to unlock the full potential of millets in Sudan and similar regions (Hassan et al., 2024).

## 11 Conclusion and future directions

Millets represent a transformative solution for tackling the modern global crises of food insecurity, environmental instability, and declining public health. In an era where shifting climate patterns increasingly threaten traditional staple crops, these hardy grains stand out as a robust alternative capable of flourishing in arid environments, high-temperature zones, and nutrient-poor soils with minimal chemical inputs. From a nutritional perspective, their high concentrations of dietary fiber, essential minerals, and bioactive molecules position them as a potent tool for addressing the “triple burden” of malnutrition, which includes undernutrition, micronutrient deficiencies, and the rapid rise of lifestyle-related chronic conditions like type 2 diabetes and cardiovascular diseases. To fully integrate these resilient crops into the global food system, a concerted effort involving strategic policy reform, targeted research funding, and robust value chain infrastructure is required.

Moving forward, the evolution of millets from underutilized “orphan crops” to mainstream staples depends on a focused, high-priority research roadmap that begins with evidence-based health validation. There is a pressing requirement for rigorous human clinical research, specifically large-scale, randomized controlled trials (RCTs), to provide definitive proof of how millet consumption influences glycemic response, insulin sensitivity, lipid profiles, and the health of the gut microbiome. Longitudinal studies focusing on specific interventions, such as finger millet or foxtail millet-based diets among pre-diabetic and diabetic cohorts, are essential to yield the clinical data needed to officially categorize these grains as functional foods with clear therapeutic properties.

In tandem with clinical research, breeding initiatives must evolve to produce millet varieties that satisfy both the farmer’s need for productivity and the consumer’s demand for nutrition. Future crop improvement strategies should prioritize the development of “nutri-cereals” that are naturally enriched with iron and zinc through biofortification, while simultaneously working to minimize antinutritional factors like phytates and tannins that can hinder mineral absorption. By leveraging advanced biotechnological tools such as CRISPR gene editing, genomic selection, and marker-assisted breeding, scientists can rapidly develop new cultivars that offer higher yields and superior resilience to environmental stress while maintaining desirable sensory qualities like improved taste and smoother texture.

To increase widespread consumer adoption, the food industry must also refine scalable processing technologies that make millets more convenient, palatable, and nutritionally accessible. Techniques such as fermentation to create probiotic beverages, malting to increase mineral bioavailability, and extrusion for healthy, ready-to-eat snacks can significantly diversify the market. Furthermore, the application of composite flour technology for products like bread and traditional flatbreads can help millets fit seamlessly into modern dietary habits. Products like fermented millet drinks and enriched bakery items have already demonstrated high consumer acceptance and improved digestibility, signaling a strong path toward successful commercialization and large-scale integration into the modern diet.

Finally, scaling the impact of millets requires a holistic strengthening of the entire supply chain, from the farm gate to the retail shelf. This involves significant investment in post-harvest management, climate-controlled storage, and streamlined supply chain logistics to ensure a steady market supply, alongside sophisticated marketing strategies like clear nutritional labeling and targeted awareness campaigns to reframe millets as premium health foods. Integrating millets into large-scale institutional programs—such as school lunch initiatives, government food assistance schemes, and hospital meal plans—can provide a guaranteed market and ensure that nutritional benefits reach the most vulnerable populations. Ultimately, a multidisciplinary synergy between scientists, breeders, technologists, and policymakers is essential to transform millets into a central pillar of a sustainable and health-oriented global food system.
